# Commentary: Using Virtual Reality to Assess Ethical Decisions in Road Traffic Scenarios: Applicability of Value-of-Life-Based Models and Influences of Time Pressure

**DOI:** 10.3389/fnbeh.2017.00247

**Published:** 2017-12-12

**Authors:** Geoff Keeling

**Affiliations:** Department of Philosophy, University of Bristol, Bristol, United Kingdom

**Keywords:** self-driving cars, moral judgements, ethical decision-making, virtual reality, robot ethics

Autonomous vehicles (AVs) will be on our roads soon. These cars will be designed so that passengers cannot take manual control in the event of a collision. These cars might encounter situations where a decision about how to allocate harm between different persons is required (Goodall, 2014; Lin, 2016). Consider,

*The Moral Design Problem:* How should manufacturers programme AVs to allocate harm in these collisions?

In a recent article, Sütfeld et al. (2017) argue that (1) human moral judgements are context dependent; such that (2) we have good reason to programme AVs to allocate harm in collisions in accordance with context-sensitive human moral judgements. Given (1) and (2), Sütfeld et al. conducted an empirical study in which participants were presented with virtual reality collisions, and data was collected on the participants' responses to these collisions. In this paper, I raise two objections to Sütfeld et al.'s approach to the moral design problem.

The first objection: Sütfeld et al.'s argument begins with the following empirical observation:

(A) Human moral intuitions about the conditions under which inflicting harm is morally permissible differ depending on context.

Sütfeld et al. take (A) as evidence for:

(B) “There is no ground truth in our ethical intuitions which holds irrespective of context.”

It is unclear how (B) should be understood. But I think the most charitable reading is that (B) is a commitment to a meta-ethical position called *particularism* (Dancy, 1983). According to *generalism*, there exists a set of normative ethical principles which determines the right thing to do in all situations. Particularism is the negation of this thesis, that is, the right thing to do is determined on a context-sensitive or case-by-case basis. The status of the evidential relation between the neuroscientific data that Sütfeld et al. use to establish (A) and meta-ethics has received little attention (Joyce, 2008). As such, it cannot be taken for granted. Given that Sütfeld et al.'s answer to the moral design problem *depends* on the plausibility of this inference, they owe an account of *why* this inference is plausible before we are justified in accepting their answer.

The second objection: let us grant that Sütfeld et al. have determined that the correct meta-ethical account is particularism. That is, the right thing to do in AV collisions must be determined on case-by-case basis. Sütfeld et al. propose to take descriptive about human snap-decisions in virtual reality collisions as an indicator of how AVs ought to be programmed in analogous contexts. Either,

(C) Sütfeld et al. are committed to the normative ethical claim that the right thing to do in AV collisions is determined by facts about human snap-judgements in analogous collisions; or
(D) Sütfeld et al. have inferred claims about how AVs *ought* to allocate harm in collisions from *descriptive* claims about how humans allocate harm in analogous collisions.

It strikes me that (D) is an invalid inference from *is* to *ought*. The fact that something is the case does not entail or suggest that it ought to be the case. This leaves us with (C). If Sütfeld et al. are committed to (C), they must explain *why* the right thing to do in driverless car collisions is determined by human snap-judgements in analogous collisions. Is this explanatory burden problematic? Here is one argument: we might reasonably expect an AV to be programmed to make *better* moral decisions in a collision than human drivers make in analogous collisions. This is not an empirical claim about how driverless cars *will be*, but instead a claim about how humans *are*. Humans are sensitive to the pressures of a collision, and under this pressure, our critical thinking capacities break-down. It is not reasonable to expect a human to make an informed moral judgement under the pressure of a life-or-death scenario. In contrast, we can reasonably expect that humans designing AV collision algorithms will not be under pressures analogous to that of a collision. So, whilst humans do not make considered moral judgements in collisions, it seems reasonable to expect an informed moral judgement from the designers of AV collision-algorithms. And if this is true, it is unclear why human snap-judgements are relevant to the moral design problem. Plausibly, we should instead use one of our best moral theories, such as utilitarianism or contractualism.

It might be objected that both Sütfeld et al. and I have set aside an important consideration: it cannot be taken for granted that AV decision-making in collisions will not evolve over time. Plausibly, AVs could be programmed with an initial collision algorithm which develops through machine-learning techniques into a more sophisticated moral decision-making algorithm over time. If this is true, the question becomes what moral principles do we programme into the AV at the beginning of the learning process. In this case, it is still unclear *why* we should take human snap-judgements as the starting principles. Moral philosophy has produced several excellent theories of moral decision-making, all of which seem like better starting points than human snap-judgements under pressure. By analogy, we might grant that AV non-moral decision-making will develop over time. As a starting point, we could either use one of our best normative theories for decision-making (e.g., expected utility theory), or programme the car to behave as humans would do in analogous circumstances. As significant thought and reflection has gone in to formulating, say, expected utility theory, it seems as though we have overwhelming reason to take it as our starting point, compared with ordinary human judgements.

In conclusion, Sütfeld et al.'s solution to the moral design problem rests on a contentious inference from neuroscientific data to meta-ethical particularism. And even granting the truth of particularism, it is unclear *why* we ought to take human snap-decisions in collisions as an indicator of how AVs ought to be programmed in analogous collisions.

## Author contributions

The author confirms being the sole contributor of this work and approved it for publication.

### Conflict of interest statement

The author declares that the research was conducted in the absence of any commercial or financial relationships that could be construed as a potential conflict of interest. The reviewer NCS and handling Editor declared their shared affiliation.
